# Universal coverage with insecticide-treated nets – applying the revised indicators for ownership and use to the Nigeria 2010 malaria indicator survey data

**DOI:** 10.1186/1475-2875-12-314

**Published:** 2013-09-10

**Authors:** Albert Kilian, Hannah Koenker, Ebenezer Baba, Emmanuel O Onyefunafoa, Richmond A Selby, Kojo Lokko, Matthew Lynch

**Affiliations:** 1 Malaria Consortium, London, UK; 2 Tropical Health LLP, Montagut, Spain; 3 Johns Hopkins Bloomberg School of Public Health Center for Communication Programs, Baltimore, MD, USA; 4 Malaria Consortium Nigeria Office, Abuja, Nigeria; 5 Malaria Consortium Africa Office, Kampala, Uganda

## Abstract

**Background:**

Until recently only two indicators were used to evaluate malaria prevention with insecticide-treated nets (ITN): “proportion of households with any ITN” and “proportion of the population using an ITN last night”. This study explores the potential of the expanded set of indicators recommended by the Roll Back Malaria Monitoring and Evaluation Reference Group (MERG) for comprehensive analysis of universal coverage with ITN by applying them to the Nigeria 2010 Malaria Indicator Survey data.

**Methods:**

The two additional indicators of “proportion of households with at least one ITN for every two people” and “proportion of population with access to an ITN within the household” were calculated as recommended by MERG. Based on the estimates for each of the four ITN indicators three gaps were calculated: i) households with no ITN, ii) households with any but not enough ITN, iii) population with access to ITN not using it. In addition, coverage with at least one ITN at community level was explored by applying Lot Quality Assurance Sampling (LQAS) decision rules to the cluster level of the data. All outcomes were analysed by household background characteristics and whether an ITN campaign had recently been done.

**Results:**

While the proportion of households with any ITN was only 42% overall, it was 75% in areas with a recent mass campaign and in these areas 66% of communities had coverage of 80% or better. However, the campaigns left a considerable intra-household ownership gap with 66% of households with any ITN not having enough for every family member. In contrast, the analysis comparing actual against potential use showed that ITN utilization was good overall with only 19% of people with access not using the ITN, but with a significant difference between the North, where use was excellent (use gap 11%), and the South (use gap 36%) indicating the need for enhanced behaviour change communication.

**Conclusions:**

The expanded ITN indicators to assess universal coverage provide strong tools for a comprehensive system effectiveness analysis that produces clear, actionable evidence of progress as well as the need for specific additional interventions clearly differentiating between gaps in ownership and use.

## Background

Until recently the two main indicators recommended for the assessment of progress in malaria prevention with insecticide-treated nets (ITN) have been the “proportion of households owning at least one ITN” and the “proportion of children under five (or pregnant women) sleeping under an ITN the previous night” [1]. These indicators have been used to monitor progress in the early years [2] as well as following the scale-up of mass distributions of ITN around 2005 [3-6] and consistently found a considerable gap between ownership of at least one ITN at household level and actual use of ITN by children which has often been interpreted as a failure to convince people to use available nets and triggered calls for better behavioural change communication (BCC) programmes [2] and/or assistance in hanging the nets [7]. While undoubtedly there is need for BCC programmes to strengthen net use and strategies have been developed for these [8], it has been pointed out as early as 2009 by Eisele and colleagues that the most important determinant of use is ownership of enough ITN, and that BCC programmes should address the gap that remains once sufficient intra-household access to ITN has been achieved [3]. This was confirmed by Hetzel *et al.* in Papua Guinea who observed that 99.5% of household members not using a net did not have access to one within the household [6] and pointed to the need to better differentiate between a lack of ITN within the household and the behavioral failure to use a net that was available.

A first step in this direction was suggested by Vanden Eng and colleagues in 2010 by proposing to categorize the population of children under five into four categories namely those living in households that: i) do not own any ITN; ii) own but did not hang an ITN; iii) own and hang but not use an ITN; and iv) those children actually using an ITN [9]. The limitation of this approach was that it still did not distinguish whether a person actually had access to an ITN within the household as categories ii) and iii) refer to non-use without confirmation that enough ITN are available. A revision of recommended indicators was then considered by the “Survey and Indicator Task Force” of the Roll Back Malaria Monitoring and Evaluation Reference Group (MERG) which in June 2011 recommended two additional core ITN indicators [10]: the “proportion of households with at least one ITN for every two people” which is considered to be enough to cover all household members [11], and the “proportion of the population that has access to an ITN within the household” and supplied detailed descriptions on how these indicators should be calculated [12]. The first indicator is to be used in conjunction with the previous indicator “proportion of households with at least one ITN” to better define the ownership gap, i.e. households with no or insufficient ITN. The second is intended to define the use gap, i.e. that part of non-use that is not explained by the absence of a usable ITN and hence open to a BCC intervention. The indicator of “access of population to ITN” has been used as a key evaluation tool in the 2012 World Malaria Report by WHO [13] and Bennet and colleagues applied the indicator of “at least one ITN for every two people” to an evaluation exercise in Sierra Leone [14]; however, in general these new indicators have not yet been broadly used.

This study applies the newly recommended indicators for the evaluation of ITN programs to the data set from the Malaria Indicator Survey of Nigeria 2010 [15] in order to explore and demonstrate their usefulness, hoping to stimulate wider utilization among malaria programme managers and public health practitioners.

## Methods

### Data set

**Figure 1:**
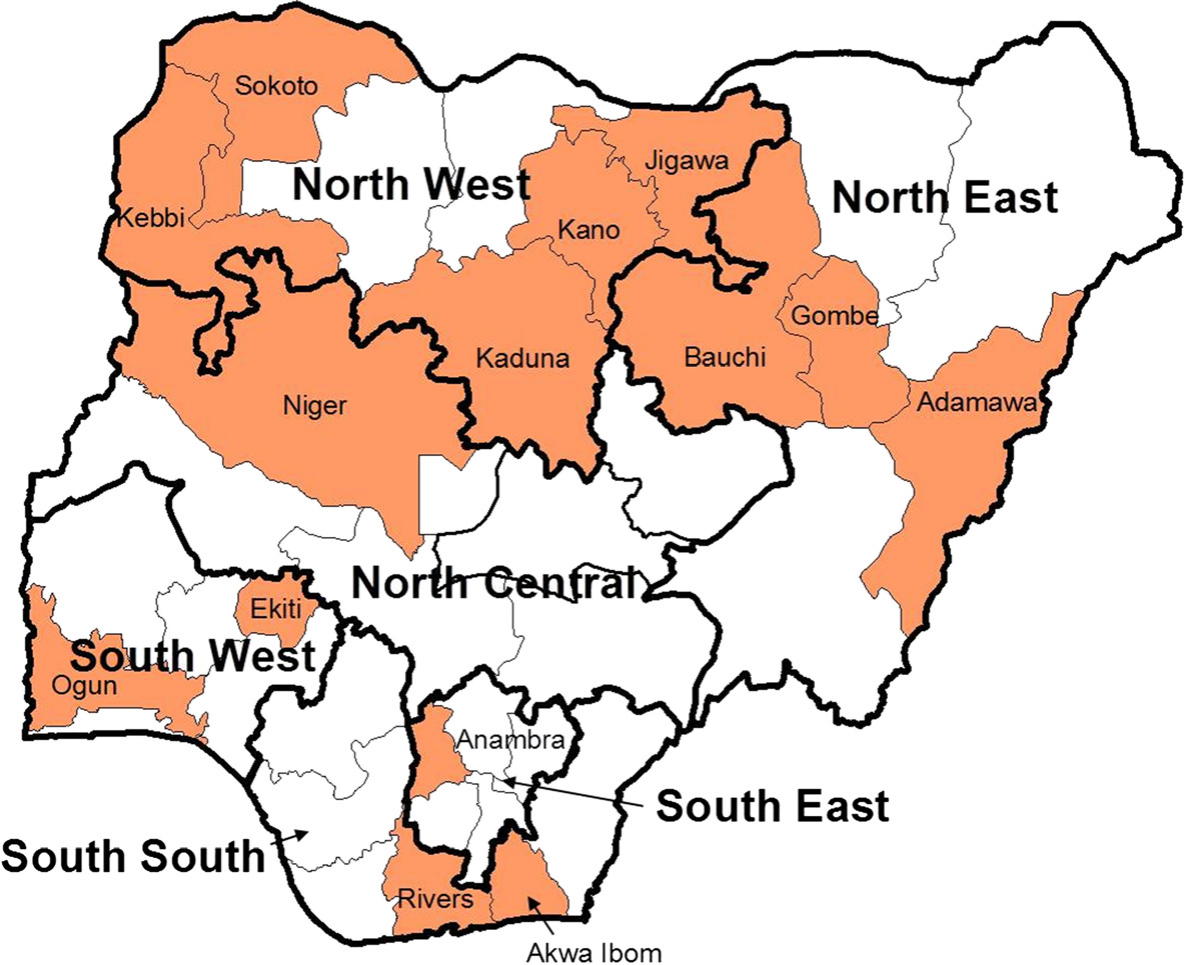
**Map of Nigeria.** Indicating the six geopolitical zones (thick border) and the States where a ITN mass campaign had already taken place before the survey (shaded areas).

The Nigeria Malaria Indicator Survey (MIS) was commissioned by the National Malaria Control Programme and carried out by the National Population Commission with support from a number of partners from the Roll Back Malaria (RBM) partnership between October and December 2010, with the objective to provide a nationally representative picture of the coverage of key malaria interventions as well as the current levels of malaria parasitaemia in children [15]. The protocol and questionnaire followed closely those recommended for MIS by the RBM Monitoring and Evaluation Reference Group [16]. The six geopolitical zones of the country (Figure 1) were defined as the sampling domains targeting 1,000 households per zone, 6,000 in total. Based on the 2006 National Population Census 40 clusters, defined as census Enumeration Areas, were selected per zone using sampling probability proportionate to population size and within each cluster 25 households were selected by simple random sampling.

The data set was downloaded with permission from the Measure DHS website and all data preparations and analyses were done using Stata 11.2 software (Stata Corp, College Station, Texas, USA).

### Calculation of indicators for universal ITN coverage

Of the four recommended indicators for assessment of universal coverage with ITN [10], two use the household as the unit of observation while the other two use the *de-facto* population, i.e. all people present in the household the night preceding the survey. The two household related indicators are:

1. *Proportion of households with at least one ITN* where the numerator consists of all households that own at least one mosquito net that was either identified as a long-lasting insecticidal net (LLIN) by the brand label or that was treated with insecticide within the past 12 months [10] and the denominator is the total of sampled households.

2. *Proportion of households with at least one ITN for every two people* where the numerator comprises all households where the ratio between number of ITN owned and the number of *de-jure* members of that household, i.e. usual members excluding visitors, is 0.5 or higher and the denominator is the total of sampled households.

The two population-based indicators are:

3. *Proportion of population with access to ITN within the household* where the numerator includes all *de-facto* household members in the sample who had access to an ITN assuming each ITN was used by two people and the denominator is the *de-facto* population in the sample. The calculation of the numerator was done in two steps as recommended by MERG [12]: first, an intermediate variable of “potential ITN users” was created by multiplying the number of ITN in each household by the factor two. In order to correct for households with more than one net for every two people the potential ITN users were set equal to the *de-facto* members in that household if the potential users exceeded the number of people in the household; second, the access indicator was calculated by dividing the potential ITN users by the number of *de-facto* members for each household and determining the overall sample mean of that fraction.

4. *Proportion of population sleeping under an ITN the previous night* where the numerator comprises all *de-facto* members identified as one of the users of an ITN based on the listings of member’s line numbers in the net roster of the questionnaire and the denominator is the *de-facto* population in the sample.

Two additional indicators were calculated to aid interpretation of the ownership and use gaps:

i. the “proportion of households with at least 1 ITN for every two people among households owning any ITN” measuring the saturation with ITN for households with any ITN. The inverse (1-p) then describes the intra-household ownership gap and can be contrasted with the spatial ownership gap, i.e. the proportion of all households that have no ITN at all.

ii. the “proportion of population sleeping under an ITN the previous night among those with access”. Because the method of calculating the access indicator does not allow allocation of access to specific individuals within the household this indicator was calculated for the overall sample or sub-groups by dividing the number of people with access, obtained by applying the weighted proportion with access to the total of the population in the sample or sub-group, by the respective *de-facto* population.. The confidence interval was obtained by first calculating the exact binomial 95% confidence interval of the proportion and then inflating it by the design effect, i.e. the ratio of the crude and adjusted 95% confidence interval for the access estimate. The inverse of this indicator (1-p) is taken as the use gap.

### Data analysis

All data analysis was done applying the sampling weights provided in the original data set and adjusting confidence intervals of estimates for the design effect by using the suite of “svy” commands in Stata. Estimates of the four relevant indicators for universal coverage with ITN were disaggregated by residence (urban–rural), wealth quintiles, the six geopolitical zones as well as a North–South grouping of these zones and whether or not the household was in a state where a mass distribution campaign had already taken place in the 18 months preceding the survey (Figure 1) based on the information provided by the National Malaria Control Programme.

The wealth index was based on household assets and obtained by principle component analysis [12] and quintiles were calculated separately for the urban and rural strata in order to adjust for differences between them. The concentration index was used as a measure of equity [17] with the value 0 representing perfect equity, +1 maximum pro-rich and −1 maximum pro-poor inequality.

Given that universal coverage with ITN aims at reduction of malaria transmission and hence a community mass effect, cluster level analysis of ITN ownership was done using a Lot Quality Assurance Sampling (LQAS) based approach as previously described by Biedron *et al.*[18] where the cluster (Enumeration Area) is considered as the “lot”. For each ITN ownership indicator the outcome of whether a defined target level of coverage was reached or not was determined by comparing the actual number of “successes” in the cluster against the decision rule. Decision rules were determined based on the target level ranging from 40% to 90% and using a 20-percentage point margin for the minimally acceptable performance (e.g. 60% for the 80% target etc.) They were obtained from the web-based ”LQAS Sampling Plan Calculator” provided by the Food and Nutrition Technical Assistance project (FANTA II) [19]. Because the sample size for each cluster was not always 25, decision rules were determined for each possible sample which ranged from 15 to 26 with 83.5% of clusters having a sample size of 24, 25 or 26.

Unless otherwise indicated statistical significance testing applied the Pearson design-based F-statistic for proportions and maximum likelihood logistic regression for multivariate analysis.

## Results

### The sample

Out of the 240 sampled clusters one was not accessible [15] so that a total of 239 clusters from all 37 states (including the Federal Capital Territory) with 5,890 households (98.2% of target) were included in the final sample. Overall 29.2% (95% confidence interval (CI) 23.3, 35.9) of households were in urban areas with a higher proportion in the South (36.8%) than in the North (22.3%, p = 0.03). The *de-jure* population in the sample was 30,336 with a mean of 4.5 per household in the North and 5.8 in the South and the *de-facto* population was 30,088 of which 22.1% were children under-five in the North and 18.0% in the South (p < 0.0001). Households headed by a female contributed 24.9% in the South and 6.7% in the North (p < 0.0001). Ownership of household assets was generally higher in the South with 76.9% owning a mobile phone, 73.4% a radio and 57.9% a television while the respective figures in the North were 44.2%, 64.5% and 23.9% respectively. Interestingly, even among the poorest wealth quintile 34.4% in the South and 15.0% in the North owned a mobile phone. A full description of the socio-economic and demographic characteristics is given in the survey report [15].

### ITN ownership

#### Type of nets owned

Among the sampled households a total of 5,169 mosquito nets were found and assessed. The vast majority of these nets were long-lasting insecticidal nets (LLIN), 95.9% in areas with the recent mass campaign (Figure 1) and 90.7% in areas without campaign. Permanet® was the most common LLIN brand (46.7%) followed by Olyset® (30.8%) while others only had a share of 3.5% to 7.0%. Only 7 nets (0.2%) were reported to have been treated with insecticide within the last 12 months and could be considered an ITN while untreated nets contributed 5.5% of the nets in the sample.

#### Source of nets

Not surprisingly, in states with a recent ITN mass-campaign 89.7% (95% CI 82.8, 94.0) of nets were obtained for free from the public sector and among these 81.5% were reported received from the campaign, 17.0% from health facilities and 1.6% from hospitals. In campaign areas 7.6% of nets were obtained from the retail market and the remaining 2.7% from private health facilities or faith-based institutions. In contrast, in the areas where the campaign was yet to be done the majority of nets (53.2%, 95% CI 42.6, 63.6) were obtained from the retail market although this was much more common in the North (69.9%, 95% CI 58.3, 79.4) than in the South (23.1%, 95% CI 17.0, 30.6, p < 0.0001). Sources were mainly open markets (88.7%) and hawkers (5.7%) while shops or supermarkets (3.5%), Patent Medicine Vendors (1.1%) and pharmacies (0.9%) played a minor role and here the pattern between North and South was similar. Even among the nets bought from the retail market 84.9% were LLIN (86.8% in the North, 72.7% in the South, p > 0.05), showing that these types of nets now dominate the market. In states without recent campaign the nets obtained from the public sector were in 62.9% from health facilities or hospital and 37.1% from previous campaigns.

Overall 10.2% (95% CI 7.9, 13.1) of households owned a net obtained from the retail market and surprisingly this rate was highest in the two poorest wealth quintiles 16.0% (95% CI 11.4, 21.8) compared to the upper three with 5.9% (95% CI 4.8, 7.2, p < 0.0001). However, the median reported price paid for an LLIN from the market increased with increasing wealth quintile from Naira 500 for the lowest two wealth quintiles to Naira 600 for the mid-quintile and Naira 800 for the wealthiest.

#### Ownership coverage

**Figure 2:**
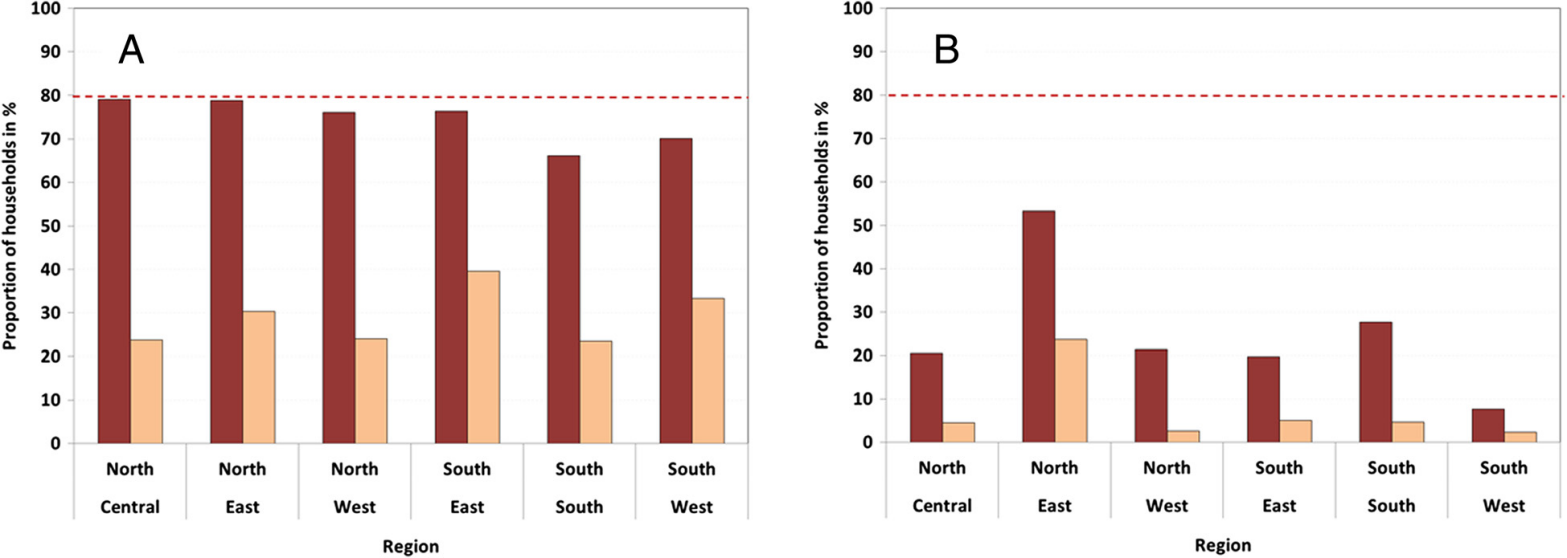
**Household ownership of ITN.** Showing ownership of at least one ITN (dark bars) and at least one ITN for every two people (light bars) for areas that did **(A)** or did not **(B)** have a recent mass campaign. Red dashed line represents the national target.

Results for the ownership indicators for ITN are presented by background characteristics in Table 1 and for geopolitical zones with and without recent campaign in Figure 2. Overall the household ownership of at least one ITN was only 42.0% with significant variations between zones and higher rates in the North (Table 1) but these differences were driven by whether or not a campaign had recently taken place in the state and this was more frequent in the North (Figure 1). In the campaign areas 74.5% of households had at least one ITN and 52.0% (95%CI 47.3, 56.6) owned at least two ITN, the target during the campaigns. In non-campaign states these rates were only 22.3% and 9.5% respectively. Considering results by zones taking into account the campaign status (Figure 2) shows that in all but the South-South (66.2%) and South-West (79.0%) the target of 80% ownership of at least one ITN was reached, while only the North-East (53.3%) showed coverage above 30% in the non-campaign areas.

**Table 1:** ITN ownership

**Background characteristic**	**Households with at least 1 ITN% (95% CI)**	**Households with at least 1 ITN for every 2 people% (95% CI)**	**Households with at least 1 ITN for every 2 people if any ITN% (95% CI)**	**Number of households**
Residence				
Urban	33.3% (26.2, 40.6)	11.5% (8.5, 15.4)	34.6% (29.7, 39.9)	1942
Rural	45.6% (39.8, 51.4)	15.3% (12.7, 18.3)	33.5% (30.2, 37.1)	3948
Zone				
North Central	32.1% (22.4, 43.4)	8.3% (4.9, 13.7)	28.9% (18.5, 34.9)	994
North East	64.5% (54.1, 73.6)	26.6% (20.7, 33.6)	41.2% (35.4, 47.3)	968
North West	58.3% (47.3, 68.5)	17.1% (12.7, 20.1)	29.3% (24.1, 35.1)	1009
South East	32.3% (23.6, 42.4)	12.7% (7.8, 20.1)	39.5% (30.3, 49.4)	997
South South	43.9% (35.5, 52.6)	12.6% (9.1, 15.8)	28.6% (23.0, 35.0)	1007
South West	21.2% (12.0, 34.6)	9.1% (5.1, 15.8)	42.8% (37.2, 48.5)	915
Region				
North	52.0% (45.5, 58.4)	17.0% (14.1, 20.4)	32.8% (29.1, 36.6)	2971
South	30.9% (25.0, 37.4)	11.0% (8.4, 14.4)	35.7% (31.5, 40.2)	2919
ITN campaign				
Yes	74.5% (70.1, 78.4)	27.2% (24.2, 30.5)	36.5% (33.0, 40.3)	2273
No	22.3% (18.8, 26.3)	6.3% (4.7, 8.4)	28.4% (23.6, 33.4)	3617
Wealth quintile				
Lowest	46.8% (38.3, 55.5)	16.6% (12.4, 21.8)	35.5% (29.6, 41.8)	1177
Second	40.9% (34.3, 48.0)	13.5% (10.7, 17.0)	33.0% (27.5, 39.1)	1178
Third	45.2% (38.9, 51.7)	16.1% (12.3, 20.7)	35.5% (29.9, 41.5)	1178
Fourth	36.0% (30.5, 41.8)	10.1% (7.7, 13.1)	28.1% (23.2, 33.5)	1178
Highest	39.9% (33.4, 46.9)	14.1% (10.7, 18.3)	35.3% (29.6, 41.4)	1179
**Total**	**42.0% (37.6, 46.5)**	**14.2% (12.1, 16.5)**	**33.8% (31.0, 36.8)**	**5890**

Distribution of any ITN ownership was very equitable with a concentration index of −0.002 (95% CI −0.016, 0.012) for areas with recent campaign and actually slightly pro-poor in areas without a recent campaign with a concentration index of −0.55 (95% CI −0.089, -0.021). The proportion of households with enough ITN for every household member, i.e. at least one ITN for every two people, was low even in the states with a recent campaign (27.2%) but again very equitable in the campaign areas, concentration index 0.035 (95% CI −0.002, 0.072), and pro-poor in the non-campaign areas, concentration index −0.184 (95% CI −0.257, -0.112).

One third of those households that owned any ITN (33.8%) also had enough ITN for all household members and this was surprisingly constant across those background characteristics included in Table 1 and varied only marginally between campaign (36.5%) and non-campaign areas (28.4%, p = 0.01). It did, however, vary significantly with household size. Overall the proportion of households with enough ITN for all was 24.5% if households size was four or less persons while only 5.1% for households with five or more members which comprised 58.1% of all sampled households (p < 0.00001). Similarly, in areas with recent campaigns 51.8% of households had enough ITN for all if there were four or less members compared to 9.5% for households with five or more (p < 0.0001).

When the ITN ownership coverage at the cluster (Enumeration Area) is considered 93.5% of the 92 clusters in states with recent campaigns were estimated to have reached at least the 50% coverage level and two-thirds (66.3%) the 80% threshold (Table 2). When the criterion of at least two ITN was applied 71.7% of the clusters reached the 50% level and only 22.8% the 80% threshold while in only 22.3% of the clusters did the proportion of households with at least one ITN for every two people reach 50%. Not surprisingly, the corresponding rates among the 147 clusters without recent campaign were very low not even reaching 20% for coverage with at least 50% of households with any ITN.

**Table 2:** ITN coverage reached at cluster (enumeration area) level

**Coverage at cluster level**	**Proportion (%) of clusters reaching coverage level**					
	**At least one ITN**		**At least two ITN**		**One ITN for every two people**	
	**No campaign**	**Campaign**	**No campaign**	**Campaign**	**No campaign**	**Campaign**
40%	26.5	97.8	8.2	83.7	6.8	39.2
50%	17.0	93.5	6.8	71.7	2.7	22.3
60%	13.6	88.0	6.1	56.2	2.0	13.0
70%	8.2	77.2	3.4	37.0	0.7	3.2
80%	5.4	66.3	2.0	22.8	0.0	0.0
90%	3.4	42.4	0.0	9.8	0.0	0.0

### ITN use

Three quarters of the nets found in the households were observed hanging over a sleeping place, 75.5% (95% CI 72.5, 78.7), and 76.8% (95% CI 73.5, 79.9) were reported as having been used the previous night of which use could be confirmed in 99.3% by the detailed list of net users resulting in a net use rate of 76.4% (95% CI 73.0, 79.4). In a multivariate logistic regression model the strongest predictor of a net being used the previous night was, not surprisingly, whether the net was hanging (adjusted Odds Ratio (OR) 40.00, 95% CI 25.70, 62.26). However, use of the net was also significantly associated with the Northern region (OR 3.30, 95% CI 2.08, 5.22), net being obtained from the retail market (OR 2.58, 95% CI 1.26, 5.26) and recent mass campaign (OR 2.16, 95% CI 1.28, 3.63) but did not vary by net type (LLIN vs. untreated), age of net or urban residence. Interestingly, net use was highest among nets from the poorest wealth quintile continuously decreasing with increasing wealth (p = 0.01 for linear trend) with an OR of 0.39 (95% CI 0.22, 0.67) for the wealthiest compared to the poorest and a concentration index of −0.080 (95% CI −0.088, -0.070).

**Figure 3:**
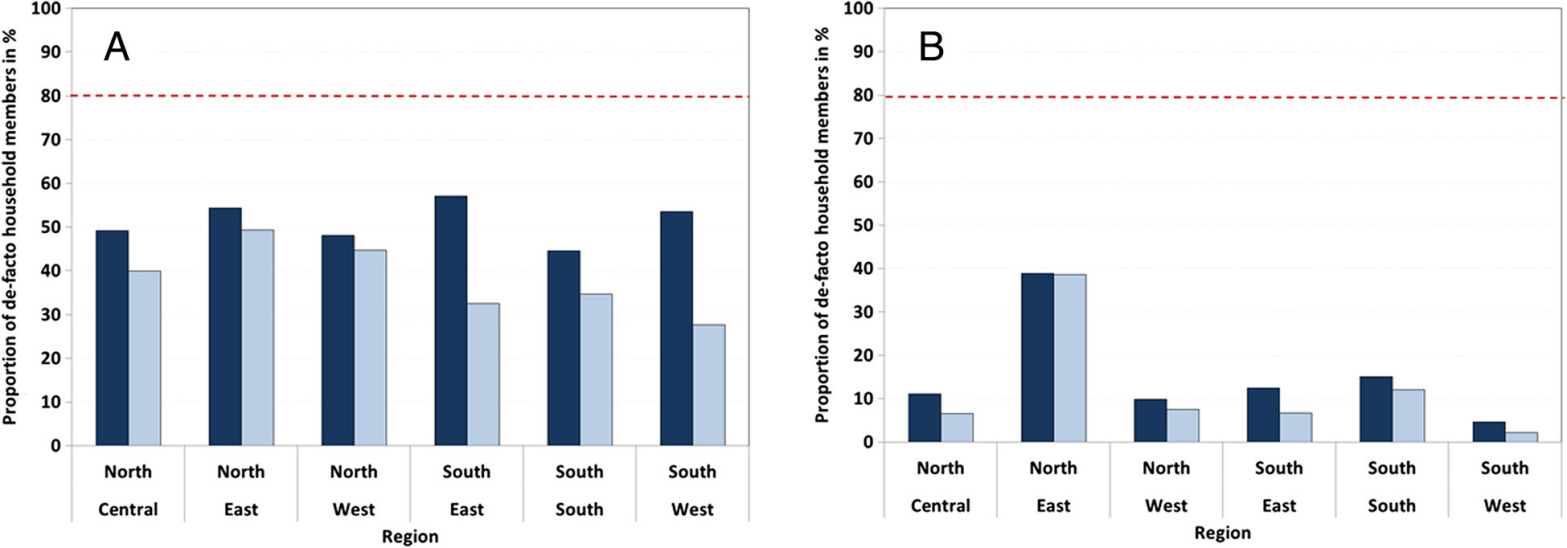
**Population access and use.** Showing access to ITN (dark bars) and use of ITN (light bars) for areas that did **(A)** or did not **(B)** have a recent mass campaign. Red dashed line represents the national target.

Net use indicators by the *de-facto* population are presented in Table 3 by background characteristics and in Figure 3 by zone and campaign status. At national level only 28.7% of the population had access to an ITN within the household and this rate increased to 50.0% in areas with a recent campaign. It did not significantly differ by wealth quintiles (p = 0.7) or urban residence (p = 0.2) and was only marginally higher in the North (p = 0.03), essentially driven by a high access rate in the North-Eastern states where no campaign had taken place (see Figure 3).

**Table 3:** Access to and use of ITN

**Background characteristic**	**People with access to ITN within household% (95% CI)**	**People who slept under ITN last night% (95% CI)**	**People who used ITN if access% (95% CI)**	**Population (*de-facto)***
Residence				
Urban	23.3% (18.5, 29.0)	16.5% (12.6, 21.4)	70.8% (59.5, 82.1)	9190
Rural	30.6% (26.5, 35.1)	25.7% (22.1, 29.8)	84.0% (77.1, 89.7)	20898
Zone				
North Central	19.8% (13.5, 28.1)	14.2% (8.9, 21.9)	71.7% (53.1, 90.3)	5171
North East	46.5% (38.7, 54.5)	43.9% (35.8, 52.3)	94.4% (88.9, 99.9)	5492
North West	34.5% (26.8, 43.0)	31.5% (24.5, 39.7)	91.3% (82.9, 99.7)	6143
South East	22.9% (16.2, 31.3)	12.7% (8.6, 18.3)	55.4% (36.3, 74.7)	4648
South South	27.3% (21.6, 33.8)	21.4% (16.8, 27.0)	78.4% (67.7, 89.1)	4927
South West	16.5% (8.7, 28.9)	8.4% (3.6, 18.3)	50.8% (17.2, 84.4)	3707
Region				
North	33.6% (28.9, 38.6)	30.0% (25.5, 34.8)	89.3% (84.0, 94.4)	16806
South	21.7% (17.2, 27.0)	13.9% (10.8, 17.6)	64.1% (58.8, 69.3)	13282
ITN campaign				
Yes	50.0% (46.7, 53.3)	41.3% (37.9, 44.8)	82.6% (79.1, 86.1)	12642
No	14.0% (11.3, 17.2)	10.9% (8.3, 14.2)	75.7% (65.4, 86.0)	17446
Wealth quintile				
Lowest	31.7% (25.0, 39.1)	29.7% (23.1, 37.3)	93.6% (87.0, 99.9)	6311
Second	28.8% (24.3, 33.7)	24.5% (20.7, 28.7)	85.1% (76.8, 93.4)	6052
Third	31.0% (26.2, 36.3)	24.9% (20.3, 30.3)	80.3% (72.2, 88.4)	6121
Fourth	25.6% (21.8, 29.9)	19.7% (16.1, 23.8)	76.9% (69.6, 84.2)	5691
Highest	25.1% (20.4, 30.4)	15.6% (12.5, 19.3)	62.1% (50.7, 73.5)	5913
**Total**	**28.7% (25.4, 32.2)**	**23.3% (20.5, 26.3)**	**81.2% (75.8, 86.6)**	**30088**

The proportion of the population actually using an ITN the previous night was 23.3%, only slightly lower than the access rate indicating a generally high level of use among those with access. Indeed, when the indicator “proportion of ITN users among those with access” is considered (Table 3), 81.2% actually used an ITN. Use among those with access was significantly higher in the North and decreased with increasing wealth quintile, from 93.6% among the poorest to 62.1% among the wealthiest, but did not differ by urban residence or campaign status.

**Figure 4:**
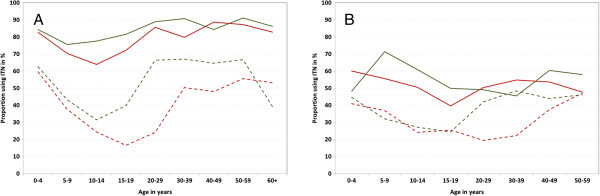
**ITN use by age, gender and ITN supply. (A)** Northern zones; **(B)** Southern zones. Solid line population from households with at least one ITN for every two people; dashed line population from households with any but not enough ITN; red male; green female.

Considering only the population from households with any ITN, use also clearly differed by age, gender and supply of ITN at household level as shown in Figure 4 separately for the Northern and the Southern zones. Except for the youngest two and the oldest age groups there was a significant gender gap in ITN use in the North for people from households with any but not enough ITN for every member with use among women much higher than among men and particularly low use rates for both at ages 10 to 19 years. For the population in households that did have enough ITN for everybody use was not only much higher ranging between 75% and 90% for women and 65% and 90% for men, but the differences by age and gender were much reduced although statistically still significant in a multivariate model (p = 0.006 for gender, p = 0.002 for age). In the South gender differences were non-existent at younger ages if not enough ITN were available but very clearly favoured women between ages 20 and 49. The most striking difference to the North was the lack of dramatic increase once enough ITN were available in the household with use rates generally not exceeding 60% even though the age curve was more straightened, i.e. there were particular gains in the least using age groups (10–19 years of age). As with the North, gender (0 = 0.01) and age (p < 0.0001) remained significant predictors of ITN use in the regression model even when sufficient ITN were available. In a joint model (North and South) of the population living in households with at least one ITN that adjusted for age, urban residence and wealth quintiles having sufficient ITN for all members was the strongest positive predictor of use (OR 3.46, 95% CI 2.88, 4.15) followed by residence in the Northern zones (OR 1.58, 95% CI 1.27, 1.98) and being female (OR 1.48, 95% CI 1.35, 1.63) while strong negative associations were found for being between age 10 and 19 (OR 0.40, 95% CI 0.36, 0.46) and belonging to the highest wealth quintile (OR 0.44, 95% CI 0.31, 0.61).

### Ownership and use gaps

Taking the inverse of the ownership and use coverage allows defining the respective gaps, i.e. the proportion of households that own no or insufficient nets and the proportion of the population that could have but did not use an ITN the previous night. These gaps are presented in Table 4 and show that although the spatial coverage gap (no ITN at all) was still significant at national level with 58.0%, it was actually quite small for those areas where a mass campaign had already been implemented (25.5%) while the intra-household gap, i.e. the level of insufficient ITN ownership among households owning any ITN was quite large (66.2%). The intra-household gap varied little across background characteristics and was only 8-percentage points higher in areas without recent campaign (63.5% vs. 71.6%, p = 0.01). However, the intensity of the intra-household gap, i.e. the number of “missing” ITN, clearly differed between campaign and non-campaign areas as households with any but not enough ITN owning on average 1.56 ITN (95% CI 1.44, 1.67) compared to 1.90 (95% CI 1.69, 2.12) in households with at least one ITN for every two people in the non-campaign areas and 1.96 ITN (95% CI 1.87, 2.05) compared to 2.20 (95% CI 2.08, 2.32) respectively in the campaign areas. In contrast, the use gap when only considering those that actually could have used an ITN was quite small overall (18.8%) and ITN utilization based on this criterion was particularly good in the North (gap 10.7%) and among the poorest wealth quintile (gap 6.4%) but low in the South (gap 35.9%).

**Table 4:** Ownership and use gaps

**Background characteristic**	**Ownership gap**		**Use gap**
	**Households with no ITN% (95% CI)**	**Households with insufficient ITN if any ITN% (95% CI)**	**People with access not using ITN% (95% CI)**
Residence			
Urban	66.7% (59.4, 73.8)	66.6% (60.1, 70.3)	29.2% (17.9, 40.4)
Rural	54.4% (51.4, 60.2)	66.5% (62.9, 69.8)	16.0% (10.3, 22.9)
Zone			
North Central	67.9% (56.6, 77.6)	71.1% (65.1, 81.5)	28.3% (9.7, 46.9)
North East	35.5% (26.4, 45.9)	58.8% (52.7, 64.6)	5.6% (0.1, 11.1)
North West	41.7% (31.5, 52.7)	70.7% (64.9, 75.9)	8.7% (0.3, 17.1)
South East	67.8% (57.6, 76.4)	60.5% (50.6, 69.7)	44.6% (25.3, 63.7)
South South	56.1% (47.4, 64.7)	71.4% (65.0, 77.0)	21.6% (10.9, 32.3)
South West	78.8% (65.4, 88.0)	57.2% (51.5, 62.8)	49.2% (15.6, 82.8)
Region			
North	48.0% (41.6, 54.5)	67.2% (63.4, 70.9)	10.7% (5.6, 16.0)
South	69.1% (62.6, 75.0)	64.3% (59.8, 68.5)	35.9% (30.7, 41.2)
ITN campaign			
Yes	25.5% (21.6, 29.9)	63.5% (59.7, 67.0)	17.4% (13.9, 20.9)
No	77.7% (73.7, 81.2)	71.6% (66.6, 76.4)	24.3% (14.0, 34.6)
Wealth quintile			
Lowest	53.2% (44.5, 61.7)	64.5% (58.2, 70.4)	6.4% (0.1, 13.0)
Second	59.1% (52.0, 65.7)	67.0% (60.9, 72.5)	14.9% (6.6, 23.2)
Third	54.8% (48.3, 61.1)	64.5% (58.5, 70.1)	19.7% (11.6, 27.8)
Fourth	64.0% (58.2, 69.5)	71.9% (66.5, 76.8)	23.1% (15.8, 30.4)
Highest	60.1% (53.1, 66.6)	64.7% (58.6, 70.4)	37.9% (26.5, 49.3)
**Total**	**58.0% (53.5, 62.4)**	**66.2% (63.2, 69.0)**	**18.8% (13.4, 24.2)**

## Discussion

The primary purpose of this study was to demonstrate the application of the recently revised and expanded indicators for universal coverage with ITN [10,12] and explore their capacity to differentiate between the lack of ITN within the communities and households referred to as ownership gap, and the inability or unwillingness to use an ITN that actually is available referred to as use gap. Based only on the previously recommended core ITN indicators of “proportion of households with any ITN” and “proportion of population (or sub-groups) using an ITN last night” [1] the results of the 2010 MIS for Nigeria show overall ITN ownership of 42% of households and 23% of the population using an ITN the previous night. When only areas with a recent mass distribution campaign of ITN are considered, 75% of households owned at least one ITN and 41% of the population used an ITN. In both cases the use estimate is only slightly more than half that of the ownership estimate suggesting a significant gap between owning and using an ITN. Such gaps have frequently been interpreted primarily as a lack of ability or willingness to use nets [2,7,20,21], although authors have also highlighted the need to clearly distinguish between the lack of nets and failure to use when available [3,6,22] both of which are included in this gap. To more precisely address the use gap some analyses have restricted data to only households owning at least one net or ITN [7,23,24] but this still leaves intra-household net density as a significant determinant [24] because some household members will still be without access. An alternative approach is to consider the net as the unit of observation [25-27] which allows a detailed look at reasons for non-use (including the physical condition of the net) but does not allow an estimate of the intra-household ownership gap at the same time. A more comprehensive evaluation framework which includes aspects of ownership and use by disaggregating the population into four categories of potential access has been suggested by Vanden Eng [9]. However, this approach only allows the identification of persons who have at least one net or ITN in their household but this does not necessarily imply that they had access if there are not enough nets for all.

In contrast, the application of the set of four ITN indicators recommended by MERG [10] and subsequent indicators of the ownership and use gaps to the Nigeria 2010 MIS data allows a much more detailed picture of the situation which clearly identifies where the successes and shortcomings are.

First, spatial coverage, i.e. proportion of households with at least one ITN, while overall only moderately high with 42%, was very close to the national target of 80% in areas that did have a mass distribution of ITN, leaving a gap if only 26% of households without any ITN (Table 4). Furthermore, using an LQAS-based assessment of each survey cluster shows that in the campaign areas almost all communities (94%) had at least 50% of households covered with at least one ITN and two thirds of the communities had an 80% coverage or better. This suggests that the target of universal spatial coverage and reduction of malaria transmission through a mass effect has been achieved by the campaigns as community level coverage as low as 50% has been shown in models to provide protection beyond the individual net users [28]. Such mass effect is likely to have significantly contributed to the reduced malaria parasitaemia prevalence observed in children in communities with a recent campaign compared to those without campaign in a secondary analysis of the Nigeria 2010 MIS data set recently presented by Kyu and colleagues [29].

Second, the analysis shows that there remained a considerable gap in the intra-household saturation with ITN, as overall 66% of households that owned any ITN did not have enough ITN for every household member, i.e. at least one ITN for every two people. This intra-household ownership gap was surprisingly constant across all background variables (Table 4) and notably did not differ much between areas with a recent campaign (63%) and no campaign (72%). However, this comparison can be somewhat misleading as the indicator only identifies the proportion of households with any gap but does not specify how big this gap is. Analysis of the mean number of ITN owned by these households showed that in campaign areas the magnitude of the gap was much smaller, with a mean of 1.96 compared to 1.56 in non-campaign areas. Nonetheless, the intra-household ownership gap remains the most critical deficit after the mass campaigns and this is caused by the NMCP strategy of limiting the number of ITN given per household to two irrespective of family size [20,29] which implied that the 58% of households in the campaign areas that had five or more members did, by definition, not get enough ITN. However, this was by design and it was clear that some additional, continuous distribution of ITN has to be established in any case to sustain universal coverage as has been emphasized by several authors [4-6,14,20] and is recommended by WHO [11]. Such continuous distribution strategies are already being implemented or piloted in Nigeria through health services, schools, the communities and the retail market (E. Baba, personal communication).

Third, using the newly recommended ITN access indicator allows differentiating in the analysis between non-use due to lack of nets and behaviour driven non-use if the person actually could have used an ITN. The results indicate that overall ITN utilization was high with only 19% of those with access not using an ITN the previous night which is very good considering that some nets are not available all nights due to washing or temporary use at other locations [22]. Furthermore, the analysis shows a clear difference between the Northern and Southern geopolitical zones in Nigeria with excellent use in the North (use gap 11%) but a considerable lack of utilization in the South (use gap 36%) implying that interventions targeting behavioural change and building of a net culture need to be strengthened in the Southern part of the country. The description of the use gap is further enhanced by the analysis of ITN use by age, gender and supply situation of the household (Figure 4) showing not only the difference between the North and the South but also that the lower use among older children and young adults which has been also found in many other settings [13,14,24] is dramatically reduced once enough ITN are available in the household, i.e. they are not primarily behaviour driven. This phenomenon has also been observed in Sierra Leone [14]. In contrast, the higher use rate of females compared to males, especially in early adulthood, which is also well described in the literature [14,24,30,31] appears to remain in Nigeria even when sufficient nets are available. This can be interpreted as a success of the campaign to emphasize the importance of women’s protection from malaria during the reproductive years.

There are two major strengths of the revised ITN indicators for the assessment of universal coverage. The first is that none of the added indicators requires changing the existing standard questionnaire modules, which allows ownership and use gaps to be easily calculated from historical data, as well as allowing analysis of trends in these specific gaps over time. The second strength is that the combination of the four indicators now allows a comprehensive analysis of malaria prevention programmes with ITN in the sense of “systems effectiveness” previously described by Tanner and colleagues [32]. This approach looks at the programme success as a cascade of dependent steps of coverage and utilization that ultimately result in the “community effectiveness”. In this case it would be the progress from spatial household ownership coverage with “at least one ITN” to the “ITN use the previous night” by the general population as seen in the combination of Figures 3 and 4. However, there is one difference to the approach originally suggested by Tanner et al. in that there is a change of denominator in the cascade from households to population making a direct comparison of these steps impossible.

There is one important limitation to the access indicator: because it is based on the ratio between number of persons and ITN at household level the indicator does not allow the determination of who among the family members has access to an ITN if there are not enough for all, meaning that comparisons between access and use cannot be done at individual level but only at the level of the overall sample or sub-groups. It also means that the calculation of the use gap estimate, i.e. the difference between access and use, and the confidence interval have to be calculated manually as suggested in this study. This, however, does not really limit the usefulness of the access indicator as a critical tool in the comprehensive analysis of the different aspects of universal coverage with ITN and the limitation can in part be overcome by using the population living in houses with sufficient ITN, who by definition should all have access to an ITN, to analyse changes in utilization by age, gender or other variables as a function of access.

The revision of the ITN indicators was initiated in 2010 and finalized in 2011. However, to date these indicators have only been broadly applied by the WHO World Malaria Report [13] and in unpublished reports from post-campaign surveys while in the more recent publications they have only partially been applied [14] or not at all [20,21,31]. Given the proven usefulness of the expanded ITN indicators there appears to be an urgent need to rapidly introduce them to a wider audience and particularly to build capacity among programme staff to appropriately utilize them.

## Conclusions

The revised and expanded ITN indicators to assess universal coverage provide researchers, managers and the public health community in general with strong tools for a comprehensive situation analysis in the sense of a “systems effectiveness” approach that produces clear, actionable evidence of progress as well as the need for specific additional interventions by clearly identifying the ownership and use gaps.

## Competing interests

The authors declare that they have no conflict of interest.

## Author’s contributions

AK, HK, ML developed the concept of the study, AK undertook the data analysis and all co-authors participated in data interpretation. Initial manuscript was drafted by AK with all co-authors contributing to final version. All authors read and approved the final manuscript.
